# Model System for the Formation of Tick-Borne Encephalitis Virus Replication Compartments without Viral RNA Replication

**DOI:** 10.1128/JVI.00292-19

**Published:** 2019-08-28

**Authors:** Wai-Lok Yau, Van Nguyen-Dinh, Elin Larsson, Richard Lindqvist, Anna K. Överby, Richard Lundmark

**Affiliations:** a Department of Medical Biochemistry and Biophysics, Umeå University, Umeå, Sweden; b Molecular Infection Medicine Sweden, Umeå University, Umeå, Sweden; c Department of Integrative Medical Biology, Umeå University, Umeå, Sweden; d Department of Clinical Microbiology, Section of Virology, Umeå University, Umeå, Sweden; University of Texas Southwestern Medical Center

**Keywords:** *Flaviviridae*, Flp-In cell line, NS4B, flavivirus, replication compartment, replication vesicles, tick-borne encephalitis virus

## Abstract

*Flavivirus* is a positive-sense, single-stranded RNA viral genus, with members causing severe diseases in humans such as tick-borne encephalitis, yellow fever, and dengue fever. Flaviviruses are known to cause remodeling of intracellular membranes into small cavities, where replication of the viral RNA takes place. Nonstructural (NS) proteins are not part of the virus coat and are thought to participate in the formation of these viral replication compartments (RCs). Here, we used tick-borne encephalitis virus (TBEV) as a model for the flaviviruses and developed a stable human cell line in which the expression of NS proteins can be induced without viral RNA replication. The model system described provides a novel and benign tool for studies of the viral components under controlled expression levels. We show that the expression of six NS proteins is sufficient to induce infection-like dilation of the endoplasmic reticulum (ER) and the formation of RC-like membrane invaginations. The NS proteins form a membrane-associated complex in the ER, and electron tomography reveals that the dilated areas of the ER are closely associated with lipid droplets and mitochondria. We propose that the NS proteins drive the remodeling of ER membranes and that viral RNA, RNA replication, viral polymerase, and TBEV structural proteins are not required.

**IMPORTANCE** TBEV infection causes a broad spectrum of symptoms, ranging from mild fever to severe encephalitis. Similar to other flaviviruses, TBEV exploits intracellular membranes to build RCs for viral replication. The viral NS proteins have been suggested to be involved in this process; however, the mechanism of RC formation and the roles of individual NS proteins remain unclear. To study how TBEV induces membrane remodeling, we developed an inducible stable cell system expressing the TBEV NS polyprotein in the absence of viral RNA replication. Using this system, we were able to reproduce RC-like vesicles that resembled the RCs formed in flavivirus-infected cells, in terms of morphology and size. This cell system is a robust tool to facilitate studies of flavivirus RC formation and is an ideal model for the screening of antiviral agents at a lower biosafety level.

## Abstract (precis)

TBEV infection causes a broad spectrum of symptoms, ranging from mild fever to severe encephalitis. Similar to other flaviviruses, TBEV exploits intracellular membranes to build RCs for viral replication. The viral NS proteins have been suggested to be involved in this process; however, the mechanism of RC formation and the roles of individual NS proteins remain unclear. To study how TBEV induces membrane remodeling, we developed an inducible stable cell system expressing the TBEV NS polyprotein in the absence of viral RNA replication. Using this system, we were able to reproduce RC-like vesicles that resembled the RCs formed in flavivirus-infected cells, in terms of morphology and size. This cell system is a robust tool to facilitate studies of flavivirus RC formation and is an ideal model for the screening of antiviral agents at a lower biosafety level.

## INTRODUCTION

Tick-borne encephalitis virus (TBEV), a neurotropic virus transmitted by ticks, accounts for one of the most important neuroinfections in adults (1). Affected areas are mainly distributed in Europe and northeastern Asia (2), and the symptoms of TBEV infection range from mild fever to severe encephalitis, which can lead to permanent damage to the nervous system (3). TBEV belongs to the genus *Flavivirus* within the family *Flaviviridae*. Many flaviviruses are pathogenic to humans, including dengue virus (DENV), yellow fever virus (YFV), Japanese encephalitis virus (JEV), West Nile virus (WNV), and the emerging Zika virus (ZIKV) (4, 5).

**FIG 1:**
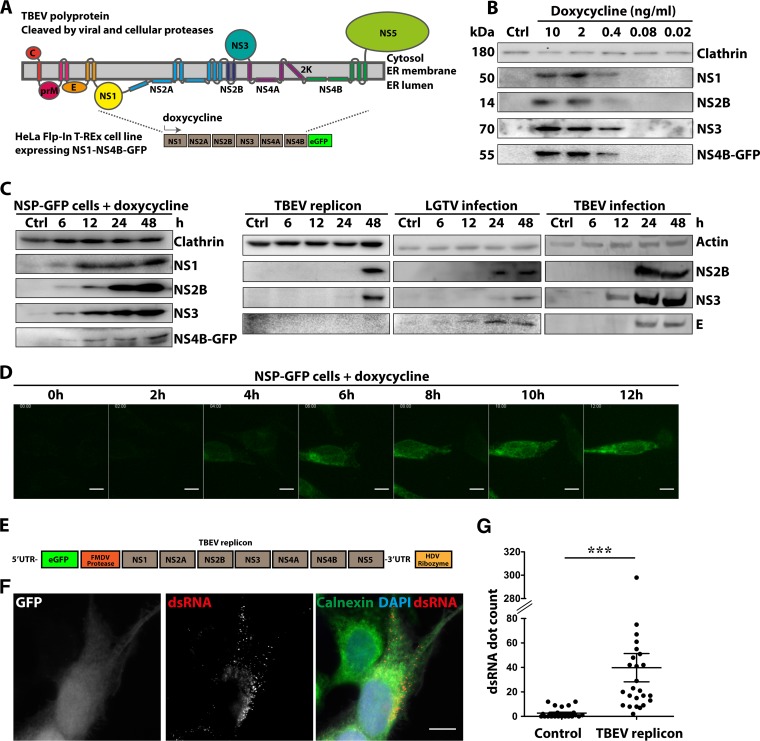
Dose- and time-dependent expression of NS1-NS4B-GFP in Flp-In T-REx HeLa cells. (A) Schematic illustration of the proposed membrane topology of the TBEV polyprotein, with color-coded representation of the individual structural (C, prM, and E) and nonstructural (NS1 to NS5) proteins. The GFP-tagged polyprotein (NS1 to NS4B) expressed following dox addition in the constructed NSP-GFP-Flp-In cells is shown below. (B) Immunoblotting expression analysis of the individual NS proteins, as indicated, following induction of NSP-GFP cells with different concentrations (0 to 10 ng/ml) of dox. Clathrin served as a loading control. (C) Immunoblotting analysis of the time-dependent expression of NS proteins, as indicated, following induction with 2 ng/ml dox in NSP-GFP cells (left), transfection with the TBEV DNA replicon (1 μg DNA per 5 × 10^6^ cells) (middle), or infection with LGTV (MOI of 1) or TBEV Torö strain (MOI of 1) (right). Actin and clathrin served as loading controls. (D) Representative fluorescence micrographs of the time-dependent expression of NS4B-GFP in NSP-GFP cells induced by 2 ng/ml dox, as recorded by live cell imaging. Scale bars = 10 μm. (E) Schematic illustration of the organization of the TBEV DNA replicon. (F) Representative images from immunofluorescence analysis of Flp-In HeLa cells transfected with the TBEV DNA replicon. Nuclei were stained with 4′,6-diamidino-2-phenylindole (DAPI), and the ER marker calnexin was labeled with specific antibody, as indicated. dsRNA was labeled with the mouse anti-dsRNA monoclonal antibody J2. The GFP signal served as the reporter for replicon transfection. Scale bar = 10 μm. (G) Quantification of dsRNA in TBEV-replicon-transfected cells (*n* = 25) and untransfected cells (*n* = 25) from the immunofluorescence analysis in panel F. The dsRNA quantification was performed with Imaris v7.5 software (Bitplane). The statistical analysis was performed with GraphPad Prism software (GraphPad Software) (*n* = 25). ***, *P* ≤ 0.01 (*t* test).

The life cycle of flaviviruses includes endocytosis into host cells, replication of the viral genome, and generation of new viral particles in which the RNA is packaged prior to cellular release (4). Viral replication and particle assembly are dependent on the expression of a viral polyprotein that is enzymatically processed to yield three structural proteins (part of the virus coat) and seven nonstructural (NS) proteins (not part of the virus coat) (Fig. 1A). The positive-sense, single-stranded RNA viral genome serves as the template for both viral RNA replication and host-mediated translation of the polyprotein. The NS proteins are involved in polyprotein cleavage and viral RNA replication, although most of their exact functions are still unclear. Viral replication is dependent on the formation of small (∼100 nm in diameter) distinct invaginations of the endoplasmic reticulum (ER) membrane (6). These so-called replication compartments (RCs) are open to the cytosol and are thought to harbor the viral RNA and a protein complex composed of the RNA-dependent RNA polymerase NS5 and other NS proteins involved in replication. Within these cavities, the viral RNA can be efficiently replicated, well hidden from the cellular response machinery specialized in detecting and degrading viral RNA (7). Simultaneously with the formation of RCs, viral coat proteins (capsid [C], premembrane [prM], and envelope [E]) form the viral budding site in conjunction with the RC in the ER (8). Subsequently, the viral RNA is transferred from the RC to the budding viral particle, which is released into the lumen of the ER and released from the cell via secretory trafficking.

The remodeling of host cell membranes into RCs is a common strategy adapted by flaviviruses and other plus-strand RNA viruses (6, 8, 9). Electron tomography has shown that TBEV induces RCs in the ER of similar type and shape as those of DENV and YFV (10). Three-dimensional (3D) modeling revealed that one-half of the RCs possessed a pore-like opening (∼10 nm in diameter) to the cytosol. In neural cells and astrocytes infected by TBEV, RCs with diameters of 60 to 90 nm have been observed (11, 12). Interestingly, the RCs in TBEV-infected neurons and astrocytes were localized either in close proximity to enveloped virions or connecting to tubule-like structures in the lumen of the rough ER (12, 13). Although the morphologies and sizes of these vesicle-like structures vary among viruses, the topology of the curvature is always the same, generating cavities open to the cytosol. The generation of such negative curvature is rare in host cells in the absence of infection. The only characterized protein complex that facilitates the generation of negative curvature is the endosomal sorting complex required for transport (ESCRT), which is known to cause budding into multivesicular bodies (14). More commonly, the interplay between proteins and lipids causes positive curvature and budding of membrane vesicles, as found for secretory and endocytic trafficking. Peripherally membrane-attached proteins and transmembrane proteins are known to generate curvature through membrane insertion, causing a wedge effect, and scaffolding, by which the protein forces the membrane to adopt the shape of the protein complex. The lipid composition also influences membrane curvature, due to the different shapes and biophysical properties of individual lipids (14). Elegant work has revealed the morphology of various types of RCs, but the architecture of protein complexes within the RCs has not been resolved.

Different viral NS proteins have been implicated in the formation of RCs (6, 15, 16). Many of these proteins have properties that may alter membrane morphology, such as multiple transmembrane domains and/or amphipathic helices (6). For example, NS1 forms dimers associated with the luminal side of the ER membrane, and recombinant NS1 remodels liposomes into lipoprotein nanoparticles (17, 18). Viral proteins may alter membrane shape directly, by associating with membranes and inducing curvature, or indirectly, by recruiting cellular factors to alter membrane morphology. A direct role for viral proteins in altering membrane curvature has been challenging to prove, however, because RC formation has not been reconstituted *in vitro* for most viruses.

Through the use of reverse genetic technology, infectious cDNA clones and subgenomic replicons of flaviviruses have been developed and widely applied to study flavivirus replication (detailed reviews are in references [Bibr B19][Bibr B20][Bibr B21]). Replicons (DNA or RNA based) are viral subgenomes without the structural protein regions used to study viral RNA replication and translation. As no infectious virus is produced, it is safe to manipulate replicon-transfected cells in biosafety level 2 (BSL2) facilities. However, the design and cloning of replicons are challenging, the system relies on RNA replication, and variations in transfection efficiency have hampered quantitative studies.

Here, we designed an inducible cell-based model system for studies of RC formation in the absence of viral RNA replication. Using this model, we can uncouple RNA replication and virion production from the membrane-remodeling activity of viral proteins during RC formation. We used TBEV as a flavivirus model and generated Flp-In HeLa cells with TBEV subgenomic DNA encoding NS1 to NS4B polyproteins stably integrated into the genome. With these cells, we can rapidly induce and titrate the expression of NS proteins to address their role in RC formation. We show that, upon addition of doxycycline (dox), NS proteins were efficiently expressed and proteolytically cleaved. The NS proteins were localized to, and formed complexes in, the ER membrane. Expression of the NS proteins without the viral RNA replication step induced the formation of dilated ER and of luminal vesicles wrapped by dilated ER, which resembled RCs in virus-infected cells. Our cell-based model system provides a robust platform to study the formation of the flavivirus replication factory and provides the possibility to identify inhibitors that target RC complex formation.

## RESULTS

### Flp-In T-REx-based model system for expression of NS proteins independent of RNA replication.

To be able to study the role of the NS proteins (NS1 to NS4B) without the influence of replication, we designed a Flp-In T-REx HeLa cell model system in which expression of the proteins NS1 to NS4B could be induced in the presence of dox. NS5 was excluded to ensure the absence of activities related to viral RNA replication. The cells were generated by introducing the last 28 amino acids from the E protein (signal sequence), followed by NS1 to NS4B of TBEV Torö strain, into the FLP recombination target (FRT) site of Flp-In T-REx HeLa cells. In order to visualize the expression, the enhanced green fluorescent protein (eGFP) reporter was fused at the C terminus of the NS4B protein (Fig. 1A). The NS-protein-expressing cells are referred to as NSP-GFP cells. Analysis of protein expression following dox addition by immunoblotting using antibodies against NS1, NS2B, NS3, and NS4B-GFP showed that the entire polyprotein was expressed and proteolytically processed into individual NS proteins of the appropriate molecular masses (Fig. 1B). Titration of the dox concentration showed that detectable protein expression was dose dependent in the range of 0.1 to 2 ng/ml dox and that the individual proteins displayed equal dose dependence. Analysis of the time dependence of protein expression by immunoblotting revealed that the processed individual NS proteins were detected already after 6 h and that expression dramatically increased up to 48 h (Fig. 1C, left). No polyprotein or unprocessed derivatives could be detected even at early time points. The time-dependent expression of NS4B-GFP could also be visualized in living NSP-GFP cells using fluorescence microscopy; the NS4B-GFP signal was detected in the perinuclear region already at 2 to 3 h and continued to increase up to 12 h (Fig. 1D).

To compare the NSP-GFP cells to previously used systems for expression of TBEV NS proteins (NS1 to NS5), we transfected HeLa cells with a TBEV DNA subgenomic replicon of the Torö strain, which requires RNA replication to provide robust viral protein expression (Fig. 1E) (22). The cytomegalovirus (CMV) promoter drives the replicon transcription of the DNA encoding NS proteins of the TBEV Torö strain. For visualization, the construct carried eGFP at the N terminus, separated from the NS proteins by an autoprotease of foot-and-mouth disease virus 2a (Fig. 1E). Immunoblotting showed that the individual proteins could be detected after 48 h following replicon transfection (Fig. 1C, middle). The delayed protein expression observed in this system is likely due to the dependence on viral RNA replication. The viral RNA replication 48 h posttransfection was detected and quantified using immunofluorescent staining with double-stranded RNA (dsRNA) antibody and was compared to that in nontransfected cells (Fig. 1F and G). The data showed that dsRNA from DNA replicon transfection could be detected in distinct puncta near or at the ER region that was stained by calnexin antibody (Fig. 1F). This finding suggested that productive RCs were formed following DNA replicon transfection. However, this system is greatly affected by low transfection efficiency. We next compared the expression of NS proteins following infection of HeLa cells with either TBEV strain Torö (multiplicity of infection [MOI] of 1) or Langat virus (LGTV) (MOI of 1), a low-virulence virus related to TBEV. Expression of NS proteins in either LGTV- or TBEV-infected HeLa cells was detected after 12 h postinfection (Fig. 1C, right). These data suggest that the NSP-GFP cells provide a robust model for controlled synchronous expression of viral proteins, independent of transfection and infection as well as viral RNA synthesis and replication.

### NS proteins form complexes in the ER.

**FIG 2:**
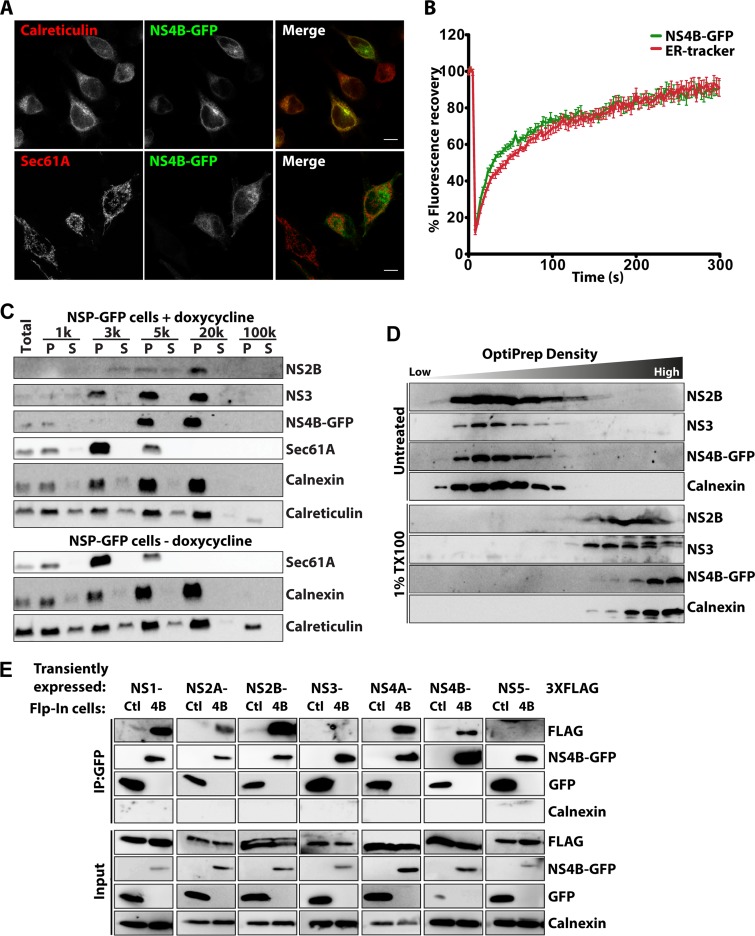
NS proteins form a protein complex in the ER of NSP-GFP cells. (A) Representative fluorescence micrographs of dox-induced NSP-GFP cells expressing NS4B-GFP and immunostained against calreticulin or Sec61A, as indicated. Scale bars = 10 μm. (B) FRAP analysis of the dynamics of NS4B-GFP in dox-induced NSP-GFP cells. ER networks were stained with ER-tracker. Regions enriched in both NS4B-GFP and ER-tracker were photobleached, and then the fluorescence signal of the regions was traced for 300 s. Means ± standard errors of the means (SEMs) are shown. (C) Immunoblot analysis of the separation of NS proteins in the supernatant (S) and pellet (P), as indicated, following lysis and differential centrifugation of induced NS1-NS4B cells at the indicated speed (1,000 to 100,000 × *g*). Antibodies to Sec61A, calnexin, and calreticulin were used as markers of the ER. (D) OptiPrep density flotation analysis of the 20,000 × *g* pellet fraction in panel C, with or without pretreatment with1% Triton-X to dissolve membranes. Flotation of calnexin and individual NS proteins, as indicated, was analyzed by immunoblotting. (E) Immunoprecipitation of GFP and NS4B-GFP from dox-induced GFP (Ctl) or NS4B-GFP (4B) Flp-In cells coexpressing FLAG-tagged NS1, NS2A, NS2B, NS3, NS4A, NS4B, or NS5, as indicated. Input and immunoprecipitated (IP) material was analyzed by immunoblotting as indicated.

To test whether NS4B-GFP localized to the ER as anticipated (23), we costained cells using antibodies against the luminal ER chaperon calreticulin and the ER translocator Sec61A after 12 h of NS4B-GFP expression. We noticed that NS4B-GFP colocalized with calreticulin but not with Sec61A (Fig. 2A), showing that NS4B-GFP was compartmentalized within ER membranes not involved in protein translation. To assay the fluidity of NS4B-GFP in ER membranes, fluorescence recovery after photobleaching (FRAP) analysis was performed. The dox-induced NSP-GFP cells were stained with ER-tracker, a dye that stains ER membrane receptors, and then photobleached. The rates of recovery of NS4B-GFP and ER-tracker were similar (Fig. 2B). We noticed that the rate of NS4B-GFP recovery was slightly greater at the early time points (0 to 100 s), which could be due to small amounts of cytosolic GFP partially cleaved from the NS4B-GFP construct.

To further validate the ER localization of NS4B-GFP, we used differential centrifugation. Analysis of the subcellular fractions showed that, while NS4B-GFP together with calnexin and calreticulin was found in the pellets from centrifugation at 5,000 × *g* and 20,000 × *g*, most of the Sec61A was pelleted during centrifugation at 3,000 × *g* (Fig. 2C). NS2B and NS3 were enriched in the same pelleted material as NS4B-GFP. Control experiments without NS protein induction showed that NS protein expression did not alter the segregation of calnexin, calreticulin, and Sec61A (Fig. 2C). Flotation analysis of the pelleted material using an OptiPrep density gradient revealed that NS2B, NS3, and NS4B-GFP were associated with membranes of a similar density as calnexin-positive membranes (Fig. 2D). When the pellet material from centrifugation at 20,000 × *g* was treated with Triton X-100, the NS-protein- and calnexin-positive membranes were disrupted and the NS proteins failed to float in OptiPrep densities (Fig. 2D). This finding confirmed that NS2B, NS3, and NS4B-GFP cosegregated into calnexin- and calreticulin-positive ER but was excluded from Sec61A-positive areas. To test whether the NS proteins interacted to form a complex with NS4B on the ER membranes, we transiently expressed the individual FLAG-tagged NS proteins in dox-induced GFP control cells or NSP-GFP cells. Cells were lysed, the membranes were solubilized with detergent, and GFP and NS4B-GFP were immunoprecipitated from the supernatant. Analysis of the cosedimented material by immunoblotting showed that all of the transmembrane NS proteins (NS2A, NS2B, and NS4A) were precipitated with NS4B-GFP but not with GFP (Fig. 2E). In contrast, of the soluble NS proteins (NS1, NS3, and NS5), only NS1 was specifically precipitated with NS4B-GFP (Fig. 2E). The ER marker calnexin was not detected in the precipitates, confirming that the membranes were solubilized and the precipitation of NS proteins with NS4B-GFP was due to physical interaction. Interestingly, FLAG-tagged NS4B was precipitated with NS4B-GFP (Fig. 2E), showing that NS4B forms stable dimers in the membrane. These data suggest that the TBEV NS proteins NS1, NS2A, NS2B, NS4A, and NS4B, but not NS3 and NS5, interact with each other and form a protein complex that is integrated into the ER membrane.

### Expression of NS1 to NS4B results in dilation of the ER and formation of RC-like invaginations.

**FIG 3:**
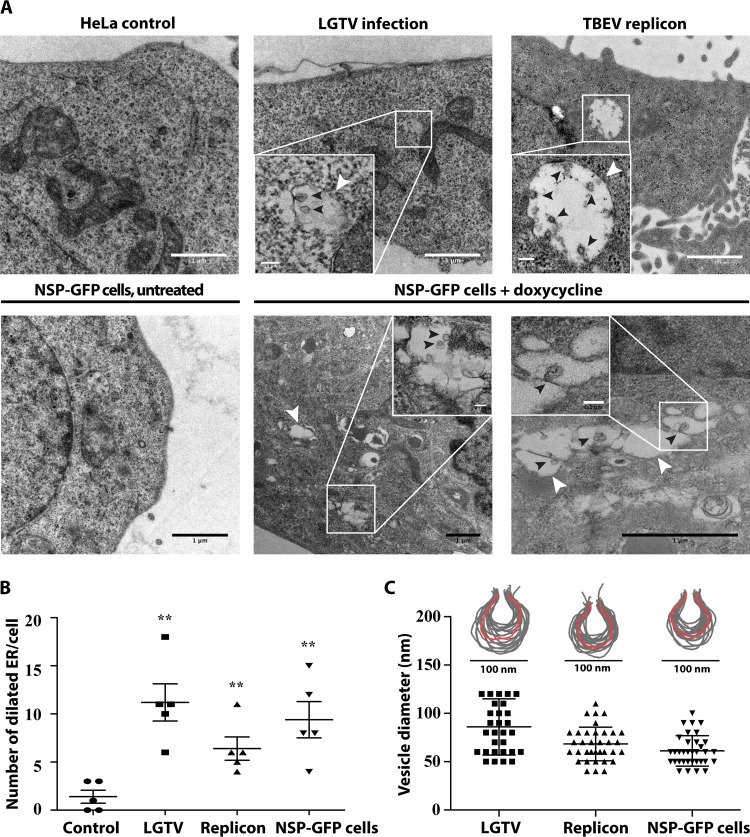
Replication-independent expression of NS1 to NS4B in HeLa cells results in dilation of the ER membrane and generation of RC-like structures. (A) Representative TEM images of control HeLa cells, cells infected with LGTV (MOI of 1), cells transfected with the TBEV DNA replicon, and uninduced and induced NSP-GFP cells. White arrowheads show dilated ER areas; black arrowheads denote replication-vesicle-like structures inside the dilated ER areas. Insets show magnifications of the indicated areas. Scale bars = 1 μm (except in insets [scale bars = 0.1 μm]). (B) Quantification of the number of dilated ER areas in replicon-transfected cells, LGTV-infected cells, and dox-induced NSP-GFP cells, compared with control HeLa cells (*n* = 5). Quantification was performed using ImageJ. **, *P* ≤ 0.05 (*t* test). (C) Membrane profiles and sizes of replication-vesicle-like structures in replicon-transfected cells, LGTV-infected cells, and dox-induced NSP-GFP cells. The membrane profiles were outlined using Adobe Photoshop. The diameters of at least 30 randomly chosen vesicles from each group were measured using ImageJ.

**FIG 4:**
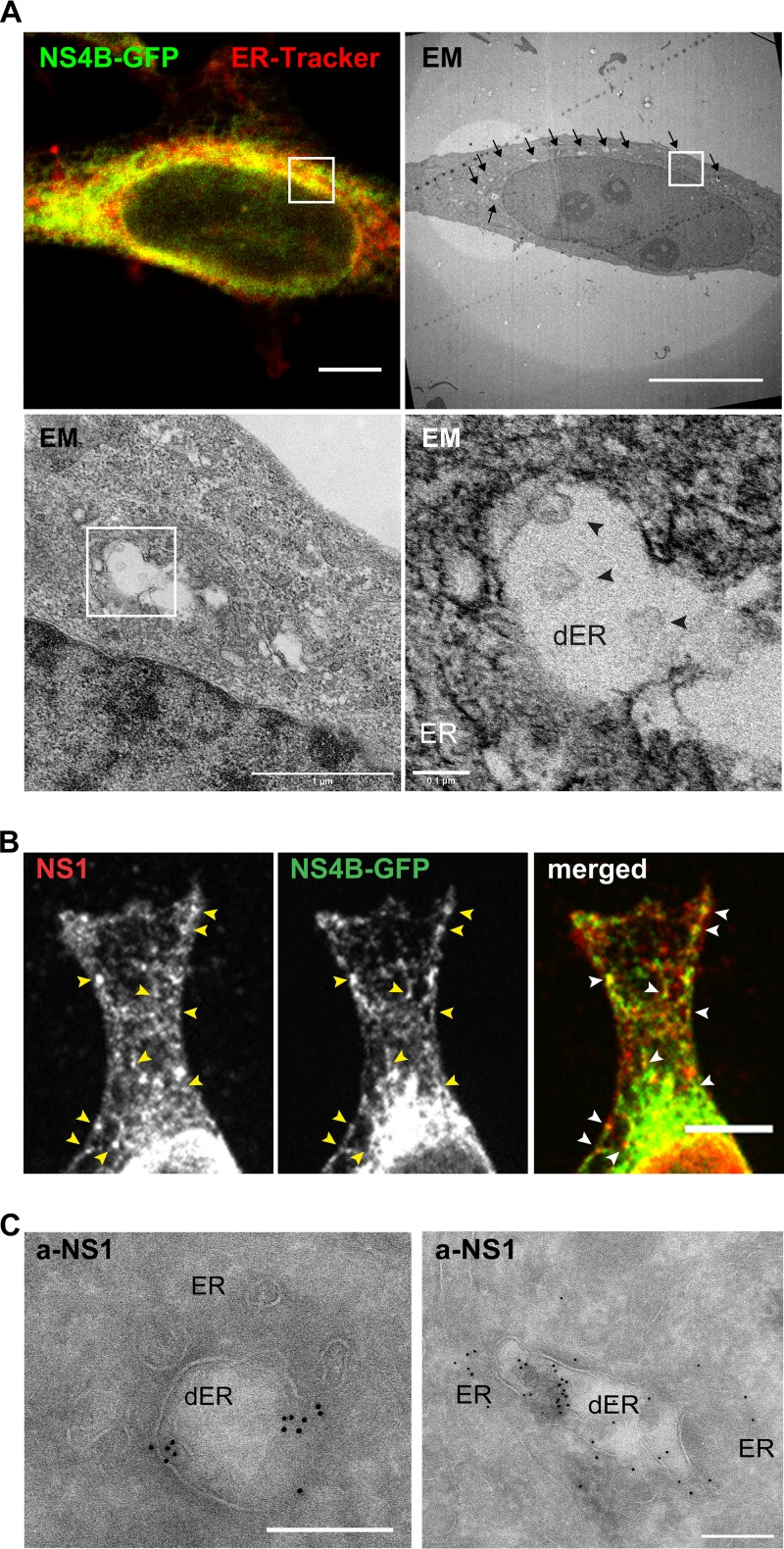
Localization of NS proteins in RC-like structures in NSP-GFP cells. (A) CLEM images of a NSP-GFP cell induced with dox. (Top) The left fluorescence micrograph shows NS4B-GFP (green) and ER-tracker (red), and the right panel shows the correlated electron micrograph, with arrows highlighting dilated ER membranes. (Bottom) The left panel shows an electron micrograph of the area indicated by the white square in higher magnification, and the right panel shows further magnification of the indicated area; the ER and dilated ER (dER) are indicated, and black arrowheads denote vesicle-like structures. Scale bars = 10 μm (top) or as indicated below the bars (bottom). (B) Immunofluorescence micrographs of the single and merged channels of a dox-induced NSP-GFP cell expressing NS4B-GFP and stained against NS1. Yellow and white arrowheads exemplify the punctuate NS1 localization on NS4B-GFP-positive ER tubules. Scale bar = 10 μm. (C) Immuno-EM images of NSP-GFP cells induced with dox. The cells were stained with anti-NS1 antibodies conjugated with gold particles. Scale bars = 200 nm.

It has been shown that TBEV causes dilation of the ER, which is a hallmark of infected cells (7). To study whether NS protein expression had an influence on membrane remodeling, the NSP-GFP cells were induced, fixed, and processed for electron microscopy (EM). As a comparison, HeLa cells were transfected with the DNA replicon or infected with LGTV. Using transmission electron microscopy (TEM), we observed frequent dilated ER compartments in the LGTV-infected cells at 48 h postinfection, which were not observed in control cells (Fig. 3A, top left). This finding was in agreement with previous work (7) and showed that HeLa cells responded similarly. Within some of the dilated ER, we observed vesicular-like invaginations reminiscent of RCs in size and shape (Fig. 3A). Some were spherical and some appeared to be connected to the ER membrane via a membrane neck. Using image analysis software, the membrane profiles of the ER-connected vesicles were determined and revealed that the sizes of the membrane bulb averaged ∼85 nm and the membrane neck ∼25 nm in diameter (Fig. 3C). Compared to the previously described infection in Vero cells (7), the invaginated structures in HeLa cells appeared more flask-shaped, were less uniform in size and morphology, and displayed a broader neck region (Fig. 3C). HeLa cells transfected with the replicon, which expressed NS5 in addition to NS1 to NS4B and relied on replication for expression but lacked the structural components, displayed the same features when analyzed (Fig. 3A). Interestingly, TEM analysis of NSP-GFP cells showed that similar types of dilated ER compartments were formed in these cells following induction (Fig. 3A, bottom). The numbers of dilated ER structures per cell detected in dox-induced NSP-GFP cells were comparable to those in LGTV-infected cells (Fig. 3B). In NSP-GFP cells, the membranes of the dilated ER compartments were frequently invaginated (Fig. 3A, bottom, insets). Analysis of the membrane profiles of these invaginations suggested that they were of overall similar morphology as the invaginations detected in replicon-transfected and LGTV-infected HeLa cells (Fig. 3C). Interestingly, the invaginations detected in NSP-GFP cells (61.18 ± 2.704 nm in diameter) and replicon-transfected cells (68.38 ± 2.861 nm in diameter) were slightly smaller than those detected in LGTV-infected cells (86 ± 5.309 nm in diameter) (Fig. 3C). To verify the correlation between the NS proteins and the dilated ER and RC-like structures, correlative light and electron tomography (CLEM) was used to resolve the ultrastructure of the NS4B-positive ER. NSP-GFP cells were induced, labeled with ER-tracker, chemically fixed, and further prepared for CLEM as described in Materials and Methods. The captured epifluorescence micrographs of NS4B-GFP and ER-tracker were overlaid with the high-resolution electron micrographs acquired from the same sample and region of interest. We found that indeed the dilated ER compartments and vesicular structures overlapped with the fluorescence of NS4B-GFP (Fig. 4A). To further confirm that the dilated ER and the vesicular structures were NSP-induced RC-like structures, we stained the NSP-GFP cells with anti-NS1 antibody. NS1 localized to discrete puncta overlapping NS4B-GFP-positive ER tubules, creating an interesting appearance like beads on a string (Fig. 4B). We interpret this as NS1 being compartmentalized in specific areas of the ER tubules. Some NS1-positive structures were devoid of NS4B-GFP, suggesting that NS1 is also trafficked independently of NS4B-GFP. In addition to the specific staining, the NS1 antibody resulted in unspecific staining of the nucleus in NSP-GFP cells and control cells (Fig. 4B and data not shown). Using Immune-gold EM analysis of NS1 localization, we found that gold particles clustered at regions of dilated ER and vesicular structures (Fig. 4C), which further supports the idea that the dilated ER structures harbor NS proteins. Taken together, these data suggest that removal of the structural components (DNA replicon) or even RNA replication (NSP-GFP cells) of TBEV is enough to cause membrane remodeling of the ER in HeLa cells.

**FIG 5:**
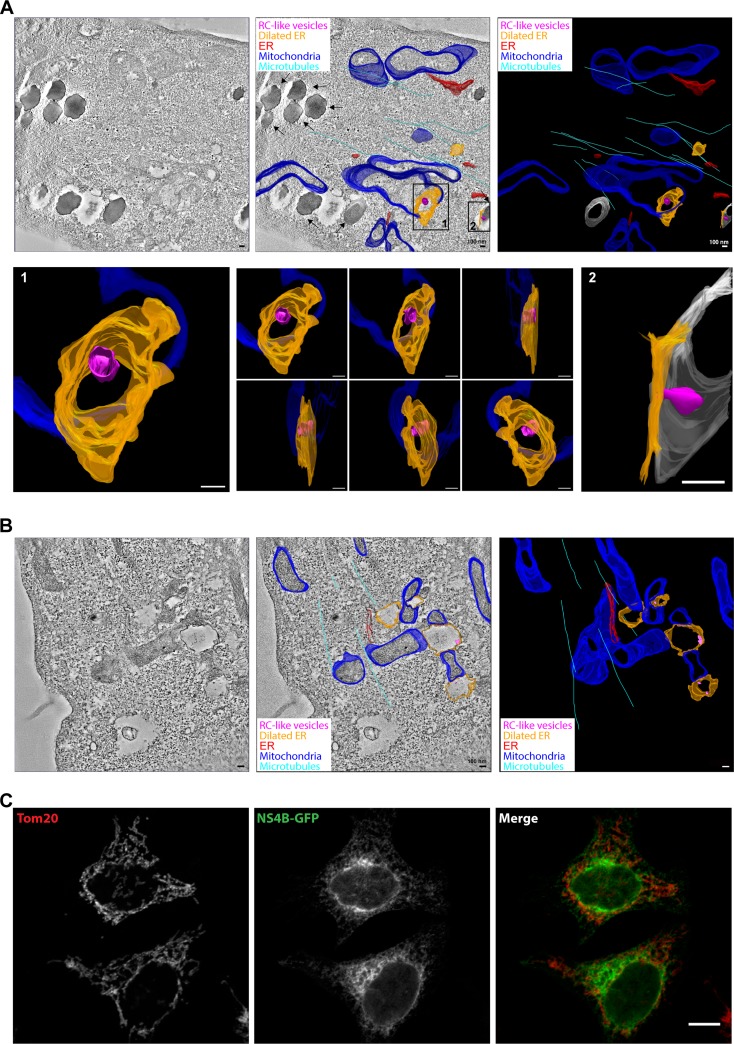
ER dilation caused by NS proteins is linked to mitochondria. (A and B) Electron tomography analysis of induced NSP-GFP cells, showing close contacts of lipid droplets, mitochondria, and dilated ER. Tomograms were recorded and the membranes of mitochondria, ER, lipid droplets, and microtubules were manually assigned, yielding a schematic, color-coded, 3D membrane model overlaid on representative tomograms. A tomographic slice (left), overlaid 3D model (middle), and 3D model (right) are presented to show dilated ER (orange) and RC-like invaginations (magenta). ER tubules are depicted in red, mitochondria in blue, microtubules in cyan, and lipid droplets in gray. Scale bars = 100 nm. (A) In the overlaid 3D model, black arrows indicate lipid droplets. Insets show magnification and different angles of the indicated areas (also see movie S2 in the supplemental material). (B) Representative electron tomograms of dox-induced NSP-GFP cells show close contact of mitochondria and dilated ER. (C) Fluorescence micrographs of NS4B-GFP in NSP-GFP cells costained for mitochondria using antibodies to the mitochondrial protein Tom20. Scale bar, 10 μm.

To more closely examine the membrane remodeling induced by expression of the NS1-NS4B proteins in the NSP-GFP cell line, NSP-GFP cells were induced and processed for electron tomography. The tomograms revealed the typical dilated ER membranes and allowed us to generate 3D models of these and other membrane compartments (Fig. 5A, top). As previously observed, many dilated ER membranes were devoid of invagination, confirming that these structures were not formed in all dilated compartments. However, more careful analysis of the detected invaginations showed that they were indeed spherical and connected to the ER membrane (Fig. 5A, bottom). From the 3D modeling, it was also apparent that both lipid droplets and mitochondrial membranes frequently formed sites of close contact with the dilated ER compartments (Fig. 5A and B; also see movies S1 and S2 in the supplemental material). Since lipid droplets originate from the ER, this might indicate that they are involved in the process of ER dilation. The close contacts of dilated ER with mitochondria suggest that also this organelle might be involved in causing the ER dilation, which is in agreement with previous studies showing the recruitment of mitochondria to viral replication sites in the ER in DENV- and ZIKV-infected cells (24, 25). Using fluorescence microscopy, we could detect NS4B-GFP-positive ER in proximity to mitochondria labeled with the marker Tom20. However, NS protein expression did not cause any apparent altered localization of mitochondria, compared to cells not expressing NS4B-GFP. Based on the data from this inducible expression model system, we propose that the expression of TBEV NS1 to NS4B causes viral protein-complex formation that is sufficient to drive ER dilation and membrane invagination of the ER in HeLa cells and that replication is not needed for this process.

## DISCUSSION

Cell lines stably expressing flavivirus replicons have facilitated the study of flavivirus RNA replication. For example, replicons have been used to study the ultrastructure of WNV RCs (26, 27) and the organization of replicated TBEV RNA in living cells (28). In this study, we have developed an inducible NSP-GFP Flp-In cell line as our model system to uncouple RNA replication and protein expression, to be able to study the direct role of NS proteins in RC formation. In this model system, the TBEV NS1- to NS4B-encoding sequence coupled to GFP is stably integrated into the Flp-In HeLa genome, under the transcriptional control of the tetracycline-regulated CMV promoter. Based on our characterization of the system, we conclude that NS proteins (i) were rapidly and homogeneously expressed and proteolytically processed, (ii) associated with the ER membrane, (iii) formed protein complexes, (iv) caused dilation of the ER membrane, and (v) mediated the formation of luminal RC-like vesicles. Therefore, we demonstrated that this inducible model system is an effective approach to study membrane remodeling, protein interactions, and RC formation.

Using fluorescence microscopy and subcellular fractionation, we showed that TBEV NS proteins (NS2B, NS3, and NS4B) were associated with ER membranes in the Flp-In system, similar to findings observed during infection. Membrane flotation analysis suggested that NS2B, NS3, and NS4B cosegregated in membranes of similar densities. These membranes were solubilized by Triton X-100 treatment, showing that the major pool of these proteins in our model system is not part of detergent-resistant domains, as previous studies have suggested (29). NS proteins are thought to form a complex in RCs, facilitating replication (6). Immunoprecipitation analysis of NS4B-GFP revealed that NS1, NS2A, NS2B, NS4A, and NS4B coimmunoprecipitated with NS4B-GFP (Fig. 2C to E), suggesting that they interact to form a protein complex. These observations are consistent with previous data on other flaviviruses. Interactions between NS1 and NS4B have been shown in both DENV and WNV, and fluorescence resonance energy transfer (FRET) analysis of recombinant WNV NS protein interactions revealed binding between NS2B and NS4B (30). Taken together, these findings suggest that the protein interactions taking place during RC formation are conserved among flaviviruses and that these can be reconstituted in our model system.

Dilation of ER with replication vesicles has been described in different cell lines infected with TBEV, including human neuronal cells, Caco-2 intestinal epithelial cells, and porcine stable (PS) kidney cells (13, 31, 32). Dilation of ER has been suggested to be linked to virion maturation and cytopathic effects of TBEV (11). Other flaviviruses, including JEV, WNV, DENV, and ZIKV, also induced dilation of the ER in infected cells (33, 34). Some plant viruses (e.g., necroviruses) also induce dilation of the ER cisternae of host cells (35). Here, we found that expression of NS1 to NS4B was sufficient to induce ER dilation in the NSP-GFP Flp-In cells in the same manner as transient transfection of the TBEV replicon and infection of HeLa cells with LGTV. We noted that the membrane of the dilated ER induced by NS proteins was smooth, without the decoration of ribosome-like structures. This finding suggests that protein localization determines that viral replication sites of plus-strand viruses are often localized at ribosome-free membranes (15). Dilation of ER cisternae may be a consequence of increased membrane phospholipid biosynthesis, which provides material to form replication vesicles or to compensate for the altered lipid homeostasis. The requirement for *de novo* lipid and membrane biosynthesis in the formation of RCs has been shown in different studies (6). Interestingly, we observed that dilated ER cisternae were frequently associated with lipid droplets, supporting the idea that dilation might be linked to lipid homeostasis. How the dilated ER correlated with the formation of replication vesicles remains to be determined, and our data show that the NS proteins drive this process independently of RNA replication and virus budding.

EM analysis revealed luminal vesicles within the dilated ER cisternae following induction of NS proteins in NSP-GFP cells. In our setup, the numbers of vesicles formed in NSP-GFP cells were similar to the numbers of RC-like vesicles formed in TBEV-replicon-transfected cells or LGTV-infected cells. However, the vesicles were slightly smaller in NSP-GFP cells and TBEV-replicon-transfected cells than in LGTV-infected cells. Compared to TBEV-induced RCs in Vero cells, the ER vesicles observed in HeLa cells (both following infection and in NSP-GFP cells) were less homogeneous in size and shape, indicating that the host cells influence the morphology of RCs. However, our data demonstrated that vesicles induced by TBEV NS1 to NS4B could be a good model to study the driving forces for formation of RCs. Differences between NSP-induced vesicles and virus-induced vesicles have been reported previously. Compared to virus-induced RCs, replicon-induced RCs are larger but fewer per ER compartment (36, 37). A similar finding was reported in a study of NS-protein-induced vesicles in which Yu et al. observed that vesicles induced by WNV NS proteins were on average larger than vesicles induced by infectious cDNA (38). This finding suggests that the size of the replication vesicles might also be affected by viral RNA. For some viruses, such as alphaviruses and nodaviruses, viral RNA in the replication complex is required for the formation of replication vesicles (39, 40). In the case of Semliki Forest virus, the size of replication vesicles might be regulated by viral RNA, which acts as a molecular ruler to define the size of the vesicles (40). Other than viral RNA, the shape of the replication vesicles might also be affected by host proteins and their abundance in different cell types. For example, tubule-like structures were observed in the dilated ER lumen of TBEV-infected nonneuronal brain cells (astrocytes) (12). In addition, luminal membrane structures were formed beside microtubules of TBEV-infected neuronal dendrites (13).

Using CLEM and electron tomography for analysis of NSP-GFP cells, we found a correlation between mitochondria and ER contact sites and NSP-induced dilation of the ER. Recruitment of mitochondria in proximity to RCs has been reported for cells infected with flaviviruses, including DENV, WNV, and ZIKV (8, 25, 41). The close proximity to RCs could increase respiration and provide more energy to support viral replication. For example, increased ATP levels were detected in hepatitis C virus replication sites and DENV-infected cells (42). Our data suggest that the recruitment of mitochondria solely induced by NS proteins may be an essential step before viral RNA replication.

In summary, our study show that the NSP-GFP Flp-In system is a simple and efficient model that allows synchronous and homogeneous expression of viral proteins in cultured cells without the need for specific biosafety facilities. Using this approach, we have uncoupled viral RNA replication from the process of membrane remodeling during the formation of RCs, and our data suggest that expression of NS1 to NS4B is sufficient to cause ER dilation and the formation of RC-like vesicles. This system will aid in mechanistic studies of membrane remodeling induced by viral proteins, as well as virus-host interactions. It could be used to screen antiviral drugs targeting membrane complex formation, and it allows studies to be conducted in BSL1 laboratories instead of BSL3 or BSL4 facilities.

## MATERIALS AND METHODS

### DNA constructs.

The TBEV (northern European strain Torö; GenBank accession no. DQ401140) subgenomic DNA replicon with eGFP as reporter was described previously (22). Expression plasmids (vector pI.18) encoding C-terminally 3×FLAG-tagged TBEV NS proteins (NS1, NS2A, NS2B, NS3, NS4A, NS4B, and NS5) were generated in a previous study (7). Vector pcDNA5/FRT/TO and Flp recombinase-encoding plasmid pOG44 (Invitrogen), used for generation of the Flp-In T-REx HeLa cell line, were obtained from Anne Simonsen, University of Oslo (Oslo, Norway).

Plasmids for generation of the Flp-In T-REx HeLa cell line expressing C-terminally GFP-tagged TBEV NS4B protein and the GFP control were constructed by standard restriction digestion-based cloning. The sequence for the 2K peptide preceding NS4B was included as a signal peptide to obtain correct topology of the membrane-spanning regions of NS4B. The DNA fragment encoding TBEV 2K-NS4B was PCR amplified with flanking 5′ KpnI and 3′ NotI restriction sites from the TBEV replicon. The amplified DNA was digested with KpnI and NotI and cloned into linearized vector pcDNA5/FRT/TO-GFP. This vector was modified to add a NotI site 5′ of the GFP-encoding region as a linker.

Plasmids for the generation of the Flp-In T-REx HeLa cell line expressing TBEV polyproteins with a C-terminal GFP tag (NSP-GFP) were assembled by the Gibson assembly method. DNA inserts, TBEV subgenomic DNA encoding the NS1-2A-2B-3-4A-4B polyprotein, and DNA encoding GFP were amplified from TBEV replicons by PCR with specific overlapping PCR primers. The vector pcDNA5/FRT/TO was linearized with the restriction enzyme BamHI. The amplified DNA inserts and the linearized vector were assembled at the complementary overlaps by using NEBuilder HiFi DNA assembly master mix (New England BioLabs), according to the manufacturer’s instructions; this yielded the plasmid pcDNA5/FRT/TO harboring TBEV subgenomic DNA encoding NS1-2A-2B-3-4A-4B-eGFP (NSP-GFP). The cloned plasmids were propagated in Escherichia coli strain Stbl3 (Invitrogen). PCR primers for amplification of the DNA inserts were ordered from Eurofins Genomics, and the sequences are available upon request.

### Cell culture, transfection, and generation of stable expression cell line.

HeLa cells (ATCC CRM-CCL-2) were grown at 37°C in 5% CO_2_ in Dulbecco’s modified Eagle’s medium (DMEM) supplemented with 10% fetal bovine serum (FBS) (EU approved, South America origin), 0.1 U/ml penicillin, and 0.1 μg/ml streptomycin. The medium, serum, and antibiotics were purchased from Gibco (Thermo Fisher Scientific). Flp-In HeLa cells hosting a FRT site in the genome were a kind gift from Stephen S. Taylor (University of Manchester, Manchester, UK). For generation of the NSP-GFP Flp-In T-REx HeLa cell line (NSP-GFP cells), Flp-In HeLa cells were cotransfected with the plasmids pcDNA5/FRT/TO (harboring TBEV subgenomic DNA encoding NS1-2A-2B-3-4A-4B-eGFP) and pOG44 using Lipofectamine 2000 (Invitrogen), according to the manufacturer's instructions. The transfected cells were grown in DMEM supplemented with 10% FBS (Gibco), 100 μg/ml hygromycin B (Invivogen), and 5 μg/ml blasticidin S (Invivogen) for plasmid selection. For inducible expression of TBEV NS proteins in Flp-In HeLa cells, doxycycline hyclate (Sigma-Aldrich) was added to the culture medium under the conditions specified.

### Virus and infection.

The TBEV strain Torö originates from an infectious clone (43); the tick-borne LGTV strain TP21 is cell culture adapted and was a kind gift from Gerhard Dobler (Bundeswehr Institute of Microbiology, Munich, Germany). The virus stocks were produced in Vero B4 cells. The Vero B4 cell monolayers were infected with LGTV or TBEV (MOI of 0.01) for 1 h at 37°C in 5% CO_2_. The virus inoculum was then removed and replaced with DMEM supplemented with 2% heat-inactivated FBS (Gibco), 0.1 U/ml penicillin (Gibco), and 0.1 μg/ml streptomycin (Gibco). The cell monolayers were further incubated for 72 h at 37°C in 5% CO_2_. The new virions in supernatant from infected Vero cells were harvested and kept as stocks at −80°C. The viral titers were determined by focus-forming assays on Vero cells, as described previously (44). These virus stocks were continued used for inoculation and titration on Flp-In HeLa cells with the same methods. The virus inoculum was then removed and replaced with DMEM supplemented with 2% heat-inactivated FBS (Gibco), 0.1 U/ml penicillin (Gibco), and 0.1 μg/ml streptomycin (Gibco). The cell monolayers were further incubated for 24 h at 37°C in 5% CO_2_.

### Antibodies and antisera.

Mouse anti-TBEV NS1 polyclonal antibody was kindly provided by Sonja Best, National Institute of Allergy and Infectious Disease. Mouse anti-TBEV E monoclonal antibody 1786.3 was a kind gift from Matthias Niedrig (Robert Koch Institute, Berlin, Germany) (45). Rabbit anti-TBEV NS2B polyclonal antiserum, chicken anti-TBEV NS3 polyclonal antibody (46), and chicken anti-TBEV NS1 polyclonal antibody (46) were produced by Agrisera (Vännäs, Sweden), using synthetic peptides (NS2B, GCVEWHPELVNEGGEVSLRVRQ; NS3, EGRDIKEFVAYASGRR) or purified NS1 protein (a kind gift from Alessandro Marcello, The International Center for Genetic Engineering and Biotechnology, Trieste, Italy) as immunogens. For NS4B-GFP fusion protein detection, the mouse anti-GFP monoclonal antibody JL-8 (Clontech) was used. For ER marker protein detection, mouse anti-calnexin monoclonal antibody AF18, rabbit anti-calnexin polyclonal antibody ab22595, mouse anti-calreticulin monoclonal antibody FMC 75, and rabbit anti-Sec61A monoclonal antibody EPR14379 were purchased from Abcam. For mitochondrial marker protein detection, mouse anti-Tom20 monoclonal antibody F-10 (Santa Cruz Biotechnology) was used. Mouse anti-actin polyclonal antibody (Cell Signaling Technology), rabbit anti-actin polyclonal antibody (Sigma), and mouse anti-clathrin heavy chain polyclonal antibody (BD Biosciences) were used for loading controls in immunoblotting. Secondary antibodies conjugated to horseradish peroxidase (HRP) (Agrisera) or conjugated to Alexa Fluor 488, 555, or 647 (Invitrogen) were used for immunoblotting detection. Secondary antibodies conjugated to Alexa Fluor 488 or Alexa Fluor 568 (Molecular Probes) were used for immunofluorescence detection.

### Immunoblotting.

Cells were lysed in sample buffer, and proteins were resolved by electrophoresis and blotted onto a polyvinylidene difluoride membrane. The membrane was probed with specific antibodies or antisera and secondary antibodies conjugated with HRP. Enhanced chemiluminescence (ECL) reagents, either the ECL Prime Western blotting detection reagent (GE Healthcare) or the SuperSignal West Femto kit (Pierce, Thermo Fisher Scientific), were used for protein detection. Chemiluminescence signals were acquired and analyzed by using the Chemidoc imaging system (Bio-Rad).

### Immunoprecipitation.

Dox-induced Flp-In T-REx HeLa cells expressing NS4B-GFP or GFP were transiently transfected with pI.18 plasmids expressing C-terminally 3×FLAG-tagged NS proteins by using Lipofectamine 2000. After 24 h of transfection, the cells were washed and scraped in cold phosphate-buffered saline (PBS). The scraped cells were lysed in lysis buffer (150 mM NaCl, 25 mM HEPES [pH 7.5], 0.5% NP-40) plus protease inhibitor cocktail (set III; Calbiochem) for 30 min on ice. The cell lysate was centrifuged at 20,000 × *g* for 10 min at 4°C to sediment insoluble cell materials. The supernatant was collected and mixed with GFP-Trap agarose beads (Chromotek) at 4°C overnight. The beads were then sedimented and washed five times with cold lysis buffer. Bound proteins were eluted by incubation at 95°C in 2× SDS sample buffer and then were analyzed by SDS-PAGE and immunoblotting.

### Subcellular fractionation and flotation analysis.

For subcellular fractionation, NSP-GFP cells were cultured in a 15-cm dish for 12 h and then treated with dox for 24 h to induce NSP-GFP expression. Cell monolayers were trypsinized and washed with cold PBS three times. The harvested cells were resuspended in 3 ml cold HEK-S buffer (15 mM HEPES-KOH [pH 7.5], 1 mM EGTA, 250 mM sucrose) and incubated on ice for 10 min. The cells were then homogenized by three passages in a ball bearing Balch homogenizer at room temperature. Cell homogenates were collected and centrifuged at 4°C at the following speeds sequentially: 1,000 × *g* for 10 min, 3,000 × *g* for 10 min, 5,000 × *g* for 10 min, and 20,000 × *g* for 30 min. The supernatant after centrifugation at 20,000 × *g* was further centrifuged at 100,000 × *g* (maximum) for 1 h at 4°C, using a Beckman MLA-130 rotor. After each centrifugation step, both the supernatant and the pellet were collected and analyzed, with the pellets being resuspended in cold HEK buffer (15 mM HEPES-KOH [pH 7.5], 1 mM EGTA).

For flotation analysis, the pellets after centrifugation at 20,000 × *g* from the subcellular fractionation experiment were resuspended in cold HEK buffer. The sample was either kept in HEK buffer or solubilized with 1% Triton X-100 (Sigma-Aldrich) in HEK buffer on ice for 30 min, with vortex-mixing. The sample (180 μl) was then mixed with 480 μl of 50% (wt/vol) OptiPrep density medium (Sigma-Aldrich) in HEK buffer, to a final OptiPrep density of 40% (wt/vol). The mixture was bottom loaded into a 4-ml polyallomer tube (Beckman Coulter). Discontinuous gradients of diluted OptiPrep (wt/vol) in HEK buffer were prepared by sequentially layering (from bottom to top) 600 μl 35% OptiPrep, 600 μl 30% OptiPrep, 600 μl 25% OptiPrep, 600 μl 20% OptiPrep, and finally 900 μl HEK buffer. The tube was then centrifuged at 160,000 × *g* (average) for 4 h at 4°C, using a Beckman SW 60 Ti rotor. After centrifugation, 300 μl from the top of the densities was collected as fraction 0, followed by fractions of 200 μl collected from the top to the bottom of the densities. The fractions (30 μl each) were incubated with 6× SDS sample buffer at 95°C and analyzed by SDS-PAGE and immunoblotting.

### Fixed and live cell imaging and quantification.

For immunofluorescence analysis, cells were fixed with 3% paraformaldehyde (PFA) in PBS for 15 min at room temperature and then washed with PBS. Fixed cells were blocked with 5% goat serum in PBS with 0.1% Triton X-100 for 3 min or with 0.05% saponin for 20 min before staining with the appropriate antibodies in PBS with 1% goat serum, using standard protocols. Confocal images of fixed cells were captured by using a Nikon A1R laser scanning confocal microscope controlled by the Nikon NIS Elements interface with a Nikon Eclipse Ti-E inverted microscope. The pictures were acquired with a Nikon CFI Plan-Apochromat 60×/1.40 Oil DIC objective, at the appropriate excitation and emission wavelengths.

For live cell imaging, cells were grown on uncoated coverslips in DMEM without phenol red (Gibco) supplemented with 10% FBS. Live cell experiments were conducted in a growth chamber (at 37°C with 5% CO_2_) connected to a Zeiss Cell Observer spinning disk confocal microscope, which was controlled by the Zeiss ZEN interface with a Zeiss Axio Observer.Z1 inverted microscope and was equipped with a CSU-X1A 5000 spinning disk unit and an iXon Ultra electron-multiplying charge-coupled-device (EMCCD) camera from Andor. Images were acquired for 13 h, at 5-min intervals, by using a Plan-Apochromat 63×/1.40 Oil DIC M27 objective, since the cells were induced with dox. Representative confocal images, time-lapse images, and videos were analyzed and prepared with ImageJ software (NIH).

For FRAP experiments, three images were acquired before regions of interest were photobleached for 1,000 ms using a 488-nm laser. Single images were than taken every 3 s after photobleaching, and recovery intensity was measured for a total of 5 min. The percentage of fluorescence recovery was calculated using GraphPad Prism software.

### TEM and quantification.

Cell monolayers were fixed with 2.5% glutaraldehyde (EM grade; TAAB) in 0.1 M sodium cacodylate buffer (CB) (pH 7.4) at 4°C overnight. The fixed cells were washed twice with CB and poststained with 1% osmium tetroxide (TAAB) in water for 1 h at room temperature in the dark. The cells were washed twice with water, followed by serial dehydration in 50%, 60%, 70%, 80%, 90%, and 100% ethanol at room temperature. The cells were then rinsed with propylene oxide, followed by infiltration in TAAB 812 resin or TAAB low-viscosity resin (TAAB) and polymerization at 60°C overnight. The embedded cells were trimmed and sectioned in 60-nm-thick sections. Samples were visualized with a JEM-1230 microscope (JEOL) operating at 80 kV. Micrographs were acquired by using a Gatan Orius 830 charge-coupled-device (CCD) camera (2,000 by 2,000 pixels; Gatan). Representative TEM images were analyzed and prepared by using ImageJ software. For quantification of dilated ER, ultrathin sections of 5 single cells from different experimental groups were randomly chosen, visualized, and quantified by using ImageJ software. The counted data were analyzed with GraphPad Prism software. The membrane profiles of replication vesicle-like structures were outlined and analyzed on TEM images by using Photoshop CC (Adobe Systems). For measurement of the sizes of vesicular structures, the diameters of 30 randomly chosen vesicles from 12 cells in each cell group were measured by using ImageJ software. The data were then analyzed with GraphPad Prism software.

### Immuno-electron microscopy.

Immunogold labeling was performed using the Tokuyasu method, essentially as described previously (47). Briefly, cells were fixed for 15 min in a BioWave Pro+ microwave system (Pelco). Ultrathin sections (80 nm) were prepared at −120°C using a Leica UC7/FC7 microtome (Leica, Wetzlar, Germany). Immunolabeling was performed for 1 h with primary antibody (chicken anti-TBEV NS1) and for 20 min with anti-chicken IgY-gold secondary antibody (Abcam, Cambridge UK). The immunogold-labeled sections were imaged using a Talos L120C transmission electron microscope (FEI, Eindhoven, Netherlands).

### CLEM and electron tomography.

NSP-GFP cells were grown on gridded glass coverslips inside glass-bottom dishes (MatTek). Dox was added to induce NSP-GFP expression for 12 h before CLEM analysis. The fixed cell monolayers were then stained with 1 μM ER-tracker red dye (Invitrogen) in CB for ER labeling and were incubated in the dark for 45 min at 37°C, after which the monolayers were fixed with 1% glutaraldehyde (EM grade; TAAB) and 3% PFA in CB for 30 min on ice. The cells were washed twice with CB and then analyzed for the fluorescence signal with confocal light microscopy. The cells of interest were located based on the grid map, and their confocal images were captured by using a Nikon A1R laser scanning confocal microscope, as described above. The TEM sample preparation of the fixed cells was then continued as described above. The cells were then infiltrated with TAAB 812 resin (TAAB), which was polymerized at 60°C overnight. The glass coverslips were removed from the embedded samples by using liquid nitrogen. The embedded samples were trimmed to locate the cells of interest and sectioned at 60 nm. The cells of interest were analyzed by using a Talos L120C microscope (FEI, Thermo Fisher Scientific) operating at 120 kV, and TEM images were acquired with a BM-Ceta CCD camera (FEI). Representative confocal and TEM images of cells of interest for CLEM were analyzed and processed by using ImageJ software and Photoshop software (Adobe Systems).

For electron tomography, the same resin that embedded NSP-GFP cells for CLEM was used for analysis. Sections of 200-nm thickness were collected on copper hexagonal grids (Ted Pella) coated with Formvar. Gold particles (10 nm) were added to the grids as fiducial markers. Single-axis tilt series (tilt range, −60° to +60°, with 1° increments) were acquired with a Talos L120C microscope operating at 120 kV and a BM-Ceta CCD camera (FEI). Three-dimensional modeling of the tomograms was performed with the IMOD v4.9.5 software package (https://bio3d.colorado.edu/imod).

## Supplementary Material

Supplemental file 1

Supplemental file 2

Supplemental file 3
