# Supplementary material for: Model System for the Formation of Tick-Borne Encephalitis Virus Replication Compartments without Viral RNA Replication
Source: J Virol. 2019 Aug 28;93(18):e00292-19. doi: 10.1128/JVI.00292-19 (PMC6714791; doi:10.1128/JVI.00292-19)
Supplement: Supplemental file 3 [file JVI.00292-19-s0003.pdf]

Supplemental material

Supplementary Movie Legends

Supplement Movie S1. Electron tomography of a pore-like opening connecting RC-like vesicles (magenta) and ER membrane (orange) in dox-induced NSP-GFP cells. Lipid droplet is depicted in grey. Scale bar = 100 nm.

Supplement Movie S2. Electron tomography of mitochondria (blue) and dilated ER (orange) in dox-induced NSP-GFP cells. RC-like vesicles (magenta), ER tubules (red), and microtubules (cyan). Scale bar = 100 nm.
